# Philosophical Reflections on Child Poverty and Education

**DOI:** 10.1007/s11217-022-09865-1

**Published:** 2023-01-12

**Authors:** Lorella Terzi, Elaine Unterhalter, Judith Suissa

**Affiliations:** 1 University of Roehampton, LDN, UK; 2 IOE, UCL’s Faculty of Education and Society, LDN, UK

**Keywords:** Poverty, Education, Capability, Deprivation, Conversion factors, Wellbeing, Inequalities, Ethical engagement

## Abstract

The harmful effects of Covid 19 on children living in poverty have refocused attention on the complex nature of child poverty and the vexed question of its relationship to education. The paper examines a tension at the heart of much discussion of child poverty and education. On the one hand, education is often regarded as essential for children’s flourishing and a means by which children can “escape” poverty; yet on the other hand, education systems, institutions, and practices, often reflect and entrench the disadvantages associated with poverty. Narratives concerning education as an escape from poverty tend not to deal in any depth with the injustices associated with poverty, stressing instead the transformative potential of education. By contrast, largely sociological analyses of the ways in which schooling reproduces inequalities tend to stop short of developing a normative account of how education can contribute to transforming the structural injustices related to poverty and its effects on children’s lives. In working to move beyond this analytic impasse, the paper shows how the cluster of concepts, which Robeyns (2018) locates as central to the capability approach, give insights which help to address these two different lacunae. The notion of conversion factors highlights the significance of taking account of existing relationships in education, while the distinction between capabilities and functionings helps guide practices regarding the education of children living in poverty. Drawing on literature on the heightened inequalities associated with poor children’s experience of lack of schooling during the COVID pandemic, the paper sketches some of the ways in which sociological analysis and normative evaluation can be linked in taking forward an “ethically engaged political philosophy” (Wolff, 2018) to discuss child poverty and education in real schools.

## Introduction

While exacerbating many of the harmful effects of poverty on children’s lives, the Covid years have brought into sharp relief the complexities of relationships of poverty and education and the ethical issues they raise. Two events exemplify this.

In January 2021, with UK schools closed due to the pandemic, children were not able to receive free meals at school, an essential support to thousands of families living in poverty. The government contracted a private company to distribute food to families receiving benefits. The food parcels were, however, of low nutritional quality and not at all plentiful. Mothers posted pictures on the internet recording the extremely meagre weekly food parcels they received in lieu of free school meals. For children living in poverty in England, the fears and disruptions of school closures associated with COVID were amplified by hunger, and carelessness of those in positions of authority distributing food. They were given a minimal handout, but indignity and exclusion were underlined.

In January 2022 Xolani Mtshali, a South African student, waiting to receive his final school leaving results, noted how impossible it had been for him to access the material distributed on TV and radio by the government when schools were shut: “*Even though I know there were TV and radio programmes for extra lessons, we do not have a TV at my house and on the radio they concentrated a lot on physics (which I did not study) and because I stay in a rural area people who would be able to help me with certain subjects stay very far from me, so it was difficult” (*Equal Education 2022*).* As the pandemic claimed millions of lives across the world it raised questions about what forms of social protection societies could offer those who were subjected to intense overt and covert discrimination. As many have noted, it also amplified the inequalities in societies (UNDP 2022; UNESCO 2022).

These two instances of the effects of Covid highlight many facets of the relationship of poverty and education indicating how the lack of resources in the households and communities of poor children, is compounded by cumulative effects of inadequate policy and practice, and experiences of social division. These incidents during the COVID pandemic prompt the need for philosophical reflection on the nature of the problem and how to address it.

Many studies of the effects of COVID, together with much of the academic work on children, education, and poverty, (e.g., Holt and Murray 2022; Hevia et al. 2022; Brehm et al. 2021), draw attention to the layers of these manifestly unjust conditions but they do not address the normative questions they raise for education. Our discussion examines this tension at the heart of much work on child poverty and education. We note how, on the one hand, education is often regarded as essential for children’s flourishing and as a means by which children can “escape” poverty. Education is thus a locus of values but the facts that shape those values are often not fully considered. Yet on the other hand, many accounts document how education systems, institutions, and practices, often reflect and entrench the disadvantages associated with poverty. In these largely sociological analyses drawing on statistical and empirical facts documenting the ways in which schooling reproduces inequalities, a number of which were assembled during COVID to record the effects of the pandemic on children’s lives (e.g., UNESCO 2021), there is a tendency for the discussion to stop short of developing a normative account of how education can contribute to transforming the structural injustices related to poverty and its effects on children’s lives. A range of facts is analysed, but the values to be addressed are often assumed more than directly articulated. In this paper we discuss problems with both these narratives. In attempting to overcome the limitations of both positions we adopt an approach of “ethically engaged political philosophy” (Wolff 2018) and seek to articulate the values and the factors that may advance the debate and inform practice. Our analysis, including a brief critical consideration of the values advanced by ideal approaches to justice, suggests that an interdisciplinary approach to these questions, informed by a normative ethical framework, can broaden out our understanding of the relationship between child poverty and education in a way that transcends the focus on the causal mechanisms by which poverty serves as a barrier to educational achievement and educational opportunities offer an “escape” from poverty.

The first part of the paper counterposes the two narratives about children’s education and poverty which arise from different perspectives and disciplinary foci, such as sociology of education and education and international development. The second part of the paper, in working to move beyond this analytic impasse associated with the two perspectives talking past each other draws on a cluster of concepts, which Robeyns (2018) locates as central to the capability approach. We use these to develop insights which help to address these two different lacunae and build some interdisciplinary framing that can help to link empirical analysis and normative evaluation in an attempt to better understand the complex interplay of child poverty and education.

## Two Narratives of Poverty and Education

The two narratives we have identified in the literature on poverty, education, and childhood, which came to be deployed in the policy discussions at the time of COVID, are firstly a narrative of children shut out of quality education by poverty, and secondly a narrative of children shut up in poor schools because of poverty. We lay out some key features of each narrative and highlight work which exemplifies this. Different disciplinary concerns shape these two narratives. While both narratives are infused with values of justice and equality, albeit considered and articulated in different domains, the different ways in which these values are positioned has implications, constraining a wider analytic engagement.

In the first narrative, poor children are locked out of the opportunities for knowledge, skills, understanding, attaining learning outcomes and qualifications, autonomy, wellbeing, and relationships of flourishing associated with formal education. In these accounts, the problem of poverty is presented as external to the school, and is associated, for example, with poor housing, run down or violent neighbourhoods, families who do not value education, or do not earn enough to pay for good education, harsh government policies or inadequate delivery of education reform, because of a poverty of ambition by officials (e.g. Barrett et al. 2019; Pritchett 2019; Azevedo 2020;). During the COVID pandemic this narrative was often deployed showing how poor girls had less access to mobile technologies than boys and missed much more schooling (UNESCO 2022).

In this narrative, schools can mitigate the effects of poverty, if they can improve their ‘quality’. Policy work drawing on this narrative emphasises that the learning outcomes of poor children are behind where they should be, giving rise to significant inequalities of educational attainment. This emphasis on the learning gap has had much attention as an outcome of schools being shut during the COVID period in the UK and internationally (Education in England 2020; World Bank 2021; House of Commons Library 2021). In these versions of this argument there is no normative question to be addressed regarding the nature of quality education, rather, the focus is mainly on a distributional issue, regarding how to improve school organisation, learning outcomes and close the attainment gap (e.g., Weidmann et al. 2021; Akmal and Pritchett 2021). Education is equated with learning outcomes in a narrow range of subjects, with little attention to a wider range of relationships, processes, experiences, and knowledge forms. Raffo et al (2009) nuance this narrative and identify the complexity of the relationships between poverty and learning outcomes, distinguishing between accounts which document forms of poverty at the micro, meso, and macro levels. They note that perspectives on education can be divided between those which are oriented to a more functionalist view of education serving society, and those which focus on how education might develop critical perspectives (Raffo et al 2009, 11–13). A mainstream version of the narrative that poverty is a problem of children out of school or learning little of value in school, we suggest, is more aligned with a functionalist view of education.

This articulates a clear perspective on the ways in which poverty or dysfunctional elements are to be kept outside the focus of education planning, which itself does not engage with the purpose of education beyond ‘learning’. This learning is generally narrowed down to a very limited number of outcomes in literacy and numeracy (Smith and Benavot 2021; D’Agnese 2017), which further contributes to the existing inequalities.

Thus, in the narrative that poverty is the problem, and education is a remedy or a way out, there is little space, firstly for understanding the perspectives and nuanced experiences of poor children, and what they and their parents say about school, and secondly for understanding the interconnection between macro, meso, and micro level political, economic, and socio-cultural relationships to produce and maintain poverty.

In contrast to the above narrative, attention to empirical research into the experience of children in poverty within educational contexts, can contribute to developing more nuanced and appropriate responses at school, community, national, and the international levels to make education a means for addressing poverty, and redistributing power. For example, Moletsane and Mitchell (2018) report on the use of participatory visual methodologies developed in partnership with poor girls in South Africa and Canada. These have demonstrated potential to contribute to ‘intensifying effort in relation to addressing the lived realities of girls who are marginalized and who suffer from persistent insecurity, injustice and abuse of power at the local level in otherwise democratic states’ (Moletsane and Mitchell 2018: 437). This analysis does not bracket poverty ‘outside’ educational experiences but looks at how a range of actors – teachers, learners, social activists—can reflexively work together to take account of its many effects and processes for change. The significance of teachers, and their positioning in this work, is noted in the work Moletsane and Mitchell have done over many years (Mitchell et al. 2020; Moletsane 2022). Recent studies of the response to COVID 19 school closures and other disasters revealed how schools in areas of socio-economic deprivation worked to offer material and practical support to children and their families in ways that went far beyond the simple requirement to ensure that poor children are able to access the formal elements of schooling (Moss et al 2020). The mobilization of school staff in deprived areas in the UK to organize food banks and supplies of basic IT equipment for families experiencing hardship during the pandemic highlighted the ways in which there were important links between education, mental health and poverty in which teachers, parents and learners’ experiences were implicated (Kim and Asbury 2020; Holt and Murray 2021; Martin et al 2022). Pre-pandemic research into the shame experienced by poor children (Chase and Bantebya-Kyomuhendo 2015) both illuminates the emotional cost of poverty and draws attention, once again, to the need to articulate a normative framework to address the complex moral demands posed by the existence of child poverty in an educational context.

If, as in the first narrative, poverty is bracketed analytically outside the school, and education is seen as a form of remedy for poverty, the analytic focus becomes how schools can compensate, rather than understanding the process of the construction of the injustices of poverty and working to enact values of justice at all levels of the educational experience.

The second narrative is associated with a wide range of work in the sociology of education (e.g., Apple 2018; Allais et al. 2019; Ball 2016). Here the analysis made is that schools reproduce poverty because of the policy frameworks and injustices of distribution inside and outside schools, the ways they interconnect, and sporadic, sometimes not well thought out ways of addressing this. The problem of poverty is inside and outside the school with significant effect on children, teachers, and families. Here there are normative questions about what constitutes a good education, and what forms of justice and obligations are required to secure it, but many of the studies do not engage with this fundamental dimension. For example, two of the key works that have had major influence on this area of scholarship—Bowles and Gintis’ study of how schools reproduce the class relationships and labour market dynamics associated with capitalism, and Pierre Bourdieu’s work on cultural capital, schooling and social reproduction – do not engage with questions of what some of the positive features of education might be (Bowles and Gintis 1976, 2002; Bourdieu and Passeron 1990).

Research into the “pedagogy of poverty” (Haberman 2010), for example, has shown how access to schooling and to material resources supporting poor children’s education is not sufficient to address the educational disadvantages they face. Such research has highlighted the ways in which children living in poverty are subject to low expectations and particular styles of pedagogical interaction on the part of educators. Hempel-Jorgensen (2019) sums up this research:

The ‘pedagogy of poverty’ experienced by learners in poverty is characterised by a focus on discipline, low intellectual engagement and a focus on attainment in tests. A study conducted in England shows how this can differ significantly in schools in which most children are not living in poverty…. This evidence suggests that English schools with low-income intakes are far more vulnerable to the pressures of high-stakes testing than schools with higher-income intakes, as children living in poverty are known to have significantly lower prior attainment. (Hempel-Jorgensen 2019 np)

Furthermore, a number of studies highlight how teachers’ misperception of poverty as caused by parents’ actions leads to negative and stereotypical behaviours which negatively affect their relationships with children (Schweiger and Graf 2015; Thompson et al. 2016; Unterhalter et al. 2012). As Treanor notes, children living in poverty express frustration at being shouted at in school, often for factors related to not having the correct equipment, which significantly affects children’s well-being, and results in progressing disengagement from learning (Treanor 2020: 83).

Several studies enhance our understanding by looking not just at the effects of poverty on children’s academic achievement, life chances, and ability to engage with pedagogical content, but at the affective significance of living in poverty (e.g., Aber et al 2007). Vizard and Hills (2015), in a comprehensive review of the social policy actions of the Conservative government in the UK from 2015 to the eve of the pandemic in 2020, note the interplay of many processes resulting in conditions for child poverty and poor educational experiences and outcomes. These include erosion of the protective capacity of the welfare state, as resource, workforce and capacity pressures across public services result in a failure to meet needs, compromising quality, and eroding the resilience of public service to shocks. Thus, the widening of inequalities across multiple axes of disadvantage set conditions for increasing poverty, which were to be accentuated during the COVID. This study highlights how it is multiple relationships in and around school that shape poverty. However, it is also distinctive for identifying a number of normative positions on top of securing adequate funding for public services, which include strengthened social rights and accountability mechanisms, such as enhancing the eroded process of democratic accountability for schooling and developing multi-dimensional strategies for change. Vizard and Hill stress giving priority to the needs of the most disadvantaged and to comprehensive public action to reduce social inequalities, together with a new values-based approach to social policy: dignity and respect, recognition and valuation (Vizard 2021). This presents a wide range of areas for normative engagement in education, made more urgent by the COVID years.

Any articulation of a normative framework for addressing child poverty as an issue of justice is clearly enhanced by a more informed and in-depth understanding of the multiple disadvantages faced by children in poverty. However, the different ways in which values are articulated and understood has implications for shaping the ethical frameworks selected.

We have highlighted how two widely circulating narratives of children, education and poverty each tend to make the normative ethical framework to understand this process somewhat fuzzy. In the next section we highlight some elements from political theory associated with the dispersed meanings of normative concern we have charted.

## Political Theory and Political Imaginaries

To summarise the above discussion, our exploration has indicated that the tension between addressing the background structural factors that create and sustain poverty and addressing the immediate educational needs of children living in poverty is a constant backdrop to the choices and judgements made by educators.

All these discussions, as Brando and Schweiger (2019: 5) note, are philosophically relevant in light of the moral questions arising from conditions of disadvantage and inequality. Normatively, ideal theories of justice, such as the prominent 1971*Theory of Justice* by John Rawls*,* provide principled positions that can guide both the analysis of what constitutes disadvantage and ways to conceptualise it, as well as principles that can guide a fair distribution of resources. Although offering also important insights for education, a thorough discussion of these is not feasible in this paper.

What can be noted, however, is that while ideal theories of justice can go some way towards offering a normative framework within which to make sense of and guide such choices and judgements, they are limited in a number of ways. Firstly, they often universalise culturally specific models of childhood, including assumptions about children’s agency and vulnerability. Within most work in liberal political theory, children are seen as young people whose capacity for moral agency in terms of self-determination, arguably an essential feature of adulthood, is not yet fully formed. They are therefore considered vulnerable and dependent, to various degrees, on the decisions and actions of adults, both within their families and other institutions, including the school. This, in turn, determines a duty of intervention to secure their material and emotional wellbeing and to defend and promote their fundamental interests, both as children and as the future adults they will become. Recent research on child poverty and education, however, questions normative positions about the moral status of children as vulnerable and dependent. Researchers have argued that such normative positions are situated in a Western and often highly abstract conception, related to the cultural and socio-economic realities of the Global North (Hanes 2019; Yasmin and Dadvand, 2019). Hanes (2019), for example, argues that in most of the Global South, children in poverty are considered autonomous and resilient agents. He also highlights how children whose life conditions and experiences do not conform to idealized conceptions of childhood suffer from marginalization and exclusion leading, in some cases, to forms of oppression, such as those seen, for example, in relation to First Nations child removal policies in Canada or the practices of transnational adoptions, whereby children from minority ethnic groups were removed from their families and respectively placed in institutions or adopted by white families (Hanes 2019: 23, 30). Thus, although different models of childhood inform all theories dealing with children, perhaps the problematic feature of highly idealized views, abstracting from empirical and contextual factors, resides in how they lead to policies that, instead of protecting children, end up further disadvantaging them.

Indeed, the very question of why we should focus on child poverty—i.e., whether the moral significance of child poverty, in and of itself, is qualitatively distinct from the moral significance of poverty—already hints at these underlying ideas about the meaning and status of childhood. Thus, child poverty as a social phenomenon can be seen to involve intersecting normative and empirical dimensions, and educational questions lie at the nexus of these different dimensions.

Secondly, theories of justice often bracket out questions about the background political structures within which socio-economic inequality and poverty are inscribed, suggesting that issues of justice are best addressed within the given political sphere through measures of redistribution and institutional reform (see Sen 2006). While we do not deny the validity of this political point, we suggest that political structures and systems are also sustained partly through a political imaginary (Taylor 2004) which legitimates and normalises certain assumptions about our social and political life. For instance, Rawlsian theories of social justice, which have significantly informed the work of both political philosophers and educational theorists concerned with educational justice and equality, assume the existence of a degree of socio-economic inequality. Relatedly, the assumption, that poverty is an inevitable part of social, economic, and political arrangements, rather than the result of political choices, is a feature of much work within the first narrative sketched above. Education systems are one of the places in which this imaginary may well be reinforced, whether through teachers’ and administrators’ attitudinal and unconscious biases, the curriculum, pedagogy, or parental choices. This is not to deny, of course, that many individuals working within these systems recognize the needs and aspirations of children living in poverty.

We have drawn attention to some weaknesses of ideal theories of justice not to reject them, but to highlight the need for such critical reflection, and to illustrate how these theories interact in complex ways when they are brought to bear on questions of how to address child poverty within an educational context (Peters and Besley 2014). Educational spaces, our analysis has suggested, are spaces in which the complexity between these different models is often most clearly illustrated and where an ethical framework is required that holds together questions of structure, agency, facts, and values. Furthermore, as the World Inequality Report (2022) states in reflecting on some of the issues that emerged during the COVID years, ‘Inequality is always a political choice and learning from policies implemented in other countries or at other points of time is critical to design fairer development pathways.'

## An Ethically Informed Perspective on Child Poverty and Education

As the discussion so far highlights, questions about the interplay of education and child poverty, brought into sharp relief by the Covid years, are best addressed with an ethically informed perspective, where the normative assumptions underlying empirical research and complex intersections of factors, including structural, cultural, and pedagogical, can be interrogated and overcome. This section shows how the cluster of concepts identified by Robeyns (2018) as central to the capability approach offers both a normative rationale for evaluating the educational disadvantage associated with poverty and insights to guide educational practice. These insights are particularly important now, in light of the widening educational inequalities resulting from the effects of the pandemic and the limited and often unsuccessful measures adopted by many countries to counteract them.

As a normative framework concerned with people’s freedom to achieve well-being,^1^ the capability approach focuses on what people can do and be with the resources they have, and what kind of life they can truly lead (Robeyns 2017: 24). At the core of the approach is a conception of well-being in terms of people’s real opportunities (capabilities) to choose among different states and activities (functionings), those that they value (Sen 4, 1992). Being a pacifist, working as a gardener or participating in the life of the community are all examples of functionings that people may value, and capability corresponds to the set of real opportunities from which they can choose (Robeyns 2017).

Normatively, the approach is used to evaluate individual well-being, relative advantage or disadvantage, the just design of institutional arrangements, as well as social policies. The distinctive contribution of the approach to these evaluations resides in considering inequalities and disadvantage in the space of capability, that is, in terms of limitations (or deprivations) of real opportunities for well-being. Questions of justice and equality, thus, are best addressed in relation to the set of opportunities available to people to lead good lives.

In addition, Sen draws attention to the importance of considering people’s different ability to make use of the resources and opportunities they have, or their conversion factors, in the evaluative exercise of justice (Sen 1992, 1999). These different factors refer to individuals’ internal features such as physical and psychological traits, as well as to factors emerging from society, for example social policies, cultural attitudes and norms, and the physical and built environment. A book, for instance, is a useful resource for reading, but not for a visually impaired person who will need a Braille version to achieve that functioning. What is important about conversion factors is that they provide information on where interventions need to be made in order to assess people’s relative disadvantage, and to expand their capabilities (Robeyns 2017). At the same time, considering these factors implies a multidisciplinary analysis, which draws on insights from different areas such as socio-economic analysis, development studies, and education, to name but a few (Robeyns 2017: 36).

The above conceptual framework brings to the fore the moral dimension of poverty, and the multiplicity of factors that contribute to it, while providing an understanding that goes beyond commonly endorsed notions of poverty in terms of lack of income and external resources (Sen 1999). As Sen notes, different sources of deprivation may compound the disadvantage associated with poverty, thus making ‘real poverty … much more intense than we can deduce from income data’ (Sen 2009: 256). And furthermore, the understanding and remedying of the persistence of poverty can ‘both be helped by explicit consideration of the relation between deprivation in different spaces, especially between incomes and the capability to lead secure and worthwhile lives’ (Sen 1992: 9). Poverty is thus defined as a deprivation of capability^2^ (Sen 2009: 254) and identified at the level of deprivation of basic functionings, such as being well nourished, sheltered, healthy and, importantly, educated. The moral urgency of addressing poverty resides therefore in the level of deprivation and deep inequality entailed, as well as in its detrimental effects on overall well-being (Burchardt and Hicks 2018).

As stated earlier, we believe that the conceptual apparatus of the approach thus outlined offers an ethically engaged rationale that brings together normative evaluation and sociological and educational analysis in addressing the disadvantage emerging from the interplay of child poverty and education, highlighted during the COVID pandemic, while going beyond idealised approaches to justice too. The notion of conversion factors highlights the significance of taking account of existing relationships in education, while the distinction between capabilities and functionings helps guide practices regarding the education of poor children. These features, as we shall see, make the approach specifically apt to counteract the aftermath of the pandemic in ways that go beyond existing measures.

We begin by noting that an understanding of educational inequalities in terms of capability limitations has the normative advantage of highlighting and considering elements of inequality that are not readily evident if we focus only on material or educational resources, such as funding (Terzi 2021). If we consider, for example, the inequalities pertaining to the experiences of schooling faced by poor children, it seems evident that no amount of additional resources, in itself, can account for the limited pedagogical interactions related to the ‘pedagogy of poverty’ which, as we have seen, can characterise poor children’s schooling. This element is best accounted for by considering the socio-cultural factors that may affect a child’s use of their educational resources and adults’ response to this. Similarly, the attitudinal and unconscious biases of teachers towards poor children cannot be appropriately addressed without considering them among the socio-cultural factors that affect children’s learning. The capability approach does not discount the importance of resources in tackling these inequalities. Rather, it draws attention to how the usefulness of resources is conditioned by a complex interaction of factors and how such complexity needs to be explicitly accounted for both theoretically and practically, in relation to appropriate interventions. Thus, the approach endorses the use of resources for additional training for teachers or public campaigns to tackle discriminating attitudes,^3^ for example. It is in this way that the process of socio-political and empirical analysis of conversion factors, which constrain or enlarge the capability space, becomes part of the normative discussion. These factors are not bracketed or treated as background, as the first educational narrative or indeed some ideal theories of justice seem to do but are part of the structuring of the normative framework. At the same time, the inclusion of these factors within a normative terrain facilitates incorporating the richness of sociological approaches to child poverty within an ethical framework allowing for acute analysis of crises like the COVID pandemic.

This is not only a theoretical evaluative approach, but one that can be translated into practice too. Many scholars working with the capability approach are able to use this core idea to incorporate multidimensionality into normative notions, and to develop more nuanced evaluative approaches to assessing social relationships in fields of education, health, and housing, to name a few (Chiappero-Martinetti, Osmani and Qizilbash 2020). As much of the extensive scholarship on the capability approach and education illuminates (e.g., Terzi 2005; Walker and Unterhalter 2007; Hart 2012; Walker 2019), the first narrative we outline, which suggests a solution of enhancing education quality inside schools, does not sufficiently attend to the complex intersecting ways in which inequality is formed both within and outside schools which the capability approach does by looking at a range of conversion factors.

The concept of conversion factors, which are environmental, political, economic, and social, highlights that the dynamics of the constraints on and expansion of capabilities entails normative choices and practical interventions. For example, a child needs literacy to enable her to access the curriculum taught at school and pass the school leaving examination. Being able to read is both a functioning and a capability. Considering environmental and political conversion factors brings to the fore how, even if a child can read, if no school has been built in her neighbourhood, or the school that is available to her is a long distance away and is staffed with teachers who lack enough experience to prepare her to enter the examination, do not talk her language, and assume children from her background are ‘backward’, the child will not be able to learn. Economic conversion factors draw attention to factors such as having to do long hours of work in the home, assisting with childcare, chores, and basic survival. Social conversion factors highlight attitudes which question, for example, a child’s school attendance because she has ‘too much book’ and does not marry young, thus incurring stigma and the risk of sexual assault. These factors need analysis both in how relationships inside and outside the school, *and their interconnection*, are understood. The two narratives we have sketched work in opposite directions and thus do not foster consideration of the processes associated with conversion factors working together. Drawing on the capability approach, in developing a framework to analyse literature on the gendered effects of school closures and return to school after COVID, a UNESCO overview report noted a wide range of conversion factors at play (UNESCO 2022).

Paying attention to conversion factors is particularly helpful in highlighting the increased inequalities of capabilities experienced as a result of Covid. As Anand et al. (2020a) highlight, while the capability of learning has been significantly affected for all children around the world, existing inequalities in access to technology, parental education, and available support have exacerbated the disadvantage of children living in poverty. Specific capabilities, such as opportunities for speech and language development have been affected, particularly in the early years and more significantly for children with special educational needs and those from the most deprived neighbourhoods (Castro-Kemp and Mahmud 2021). As noted above, these inequalities are the result of complex interconnected environmental, social, political, and cultural factors, which are foregrounded and assessed by the capability approach. Relatedly, the opportunities to make up for the so called ‘learning loss’ caused by Covid differ too. And while some governments have attempted to address the learning loss by providing additional funding for specific programmes, for instance additional one to one and small group lessons or extended school time, such as the National Tutoring Programme deployed in England, the underlying structural social, economic, and educational inequalities have remained unchanged or have worsened, thus leading to the potential limited success of such initiatives. Consider, for example, the case of Janice, a year 7 student with insecure literacy skills attending a school in a poor neighbourhood. Janice had problems accessing remote learning and her learning has suffered as a result; moreover, she does not qualify for free access to school meals and often arrives at school hungry. Although teachers’ intervention is crucial in providing Janice with appropriate learning support, it is questionable whether such support will work, if the circumstantial factors of Janice’s situation are not addressed, and, importantly, the teaching and learning are based on limited notions of ‘catching up’. Through its attention to conversion factors, we suggest, an approach based on capability draws much needed attention to the role of schools in addressing disadvantage in interrelation with policies addressing circumstantial factors.

Finally, we posit, the conceptual apparatus of the capability approach, and its distinction between functionings and capability, offers insights that may prove helpful for addressing the education of children living in poverty. The importance accorded by the approach to well being and to the opportunities to lead good lives suggests, first and foremost, a shift from a functionalist view of education and its emphasis on a narrow set of learning outcomes, to a view of education aimed at the expansion of children’s capabilities. This more expansive view of education could, perhaps, include an expansion of the social imaginary associated with the dominant normative frameworks that we have outlined above. Thus, education is recognised as having a fertile role in enhancing children’s present and future well-being and providing real opportunities to develop functionings that will enable them to participate in the life of their communities and their broader society, and in work and leisure activities (Nussbaum 2011). At the same time, education itself can be one of the ways in which possibilities for social change can be imagined. A number of accounts using the capability approach to analyse educational relationships highlight this (e.g., De Jaeghere 2021; Walker et al. 2022; Unterhalter et al. 2022). While not discounting the importance of achieving functionings pertaining to, e.g., literacy and numeracy, the approach suggests instead a much broader conception of education, one more akin to promoting the full functionings pertaining to local, national, and global citizenship and the capacity to reflect on one’s values and goals and those of the surrounding society.

Moreover, the broader scope of education in relation to the expansion of capabilities is antithetic to any form of reductionist pedagogy, such as the pedagogy of poverty outlined earlier, but supports instead forms of pedagogical interaction and co-construction of the learning and teaching process (Brando 2020; Adamson 2021).

Lastly, the focus on capabilities, at least at the level of policy, helps unmask the inequalities of children living in poverty by considering the real opportunities they have, beyond the actual level of functionings or learning outcomes they may achieve.

It can be seen that the capability approach provides insights that allow us to address the lacunae identified in the two narratives of childhood, poverty and education we have sketched, and which continue to inform policy after the Covid years. In the first narrative the space of education is conceived as separate from the contexts of poverty, while in the second narrative the space of education is submerged by the structures associated with the perpetuation of poverty. The capability approach identifies the space of capabilities as different to these conceptions. Education has the potential to be a space of capabilities and opportunities that is not separated from social conditions, as the approach acknowledges the salience of conversion factors. But the capability space is also not simply a reflection of what may be the narrow set of opportunities offered by a society that is structured to reproduce inequalities. The capability space may be understood as a site of agency and opportunity contoured by the many historical and contemporary conditions that shape child poverty.

An ethical framework informed by the conceptual apparatus of the capability approach shows how values and ideals informed by the invaluable insights provided by educational, sociological and empirical studies can contribute to an understanding of the complexity of child poverty and the role that education plays in children’s life.

## Conclusion

The Covid 19 pandemic has starkly refocused attention to the pressing and growing situation of children living in poverty worldwide. Capabilities threatened during the pandemic include opportunities for health, wellbeing, equity and inclusion. Work on the Covid pandemic and the capability approach has prompted a rich investigation of the ways in which institutions and innovations should be approached (Venkatapuram 2020; Anand et al. 2020; Ferrannini et al 2021; UNDP 2022). Our discussion has explored two dominant narratives about child poverty in its interplay with education and it has highlighted how each overlooks the complex ways in which normative values and contextual factors are related. The interdisciplinary conversations that enable theorists and practitioners to develop adequate responses to the pressing reality of child poverty, which has been starkly emphasised by the recent Covid 19 pandemic, we have argued, demand that we adopt an ethically informed, normative position on the desirability and the feasibility of intervening in our educational institutions, in ways that promote people’s real possibilities for leading a flourishing life. The modest, but also the possible radical potential of this perspective is captured in Jonathan Wolff’s suggestion that, at least in principle, ‘it is possible to improve individuals’ opportunities, and hence their capability set, by doing any of three interventions: improving a person’s internal traits such as strengths, skills etc., or improving their external resources including income, entitlement to services etc., and finally improving the social and cultural environment in which a person lives, including social policies, cultural attitudes and norms as well as their built environment’ (Wolff, 2007, Wolff, 2020). The normative framework we have articulated is perhaps a first step towards such an improvement on the existing situation.

## Footnotes

1 Amartya Sen originally formulated the approach as an alternative to predominant accounts of well-being based on utility (Sen 1985, 1992), while Martha Nussbaum further articulated it through a list of ten central capabilities deemed necessary to live a truly human life (2000, 2010).

2 Sen’s position has however been critiqued for obscuring material deprivation as fundamental to poverty. See Lister (2004), for an articulated discussion.

3 We are grateful to an anonymous reviewer for pressing on this point.

## References

[CR1] Aber, J. et al. (2007). *Poverty and child development: new perspectives on a defining issue*. 10.1037/11486-009

[CR2] Adamson L (2021). Language of instruction: A question of disconnected capabilities. Comparative Education.

[CR3] Akmal M, Pritchett L (2021). Learning equity requires more than equality: Learning goals and achievement gaps between the rich and the poor in five developing countries⋆. International Journal of Educational Development.

[CR4] Allais S, Cooper A, Shalem Y (2019). Rupturing or reinforcing inequality? The role of education in South Africa today. Transformation.

[CR5] Anand P, Ferrer B, Gao Q, Nogales R, Unterhalter E (2020). COVID-19 as a capability crisis: Using the capability framework to understand policy challenges. Journal of Human Development and Capabilities.

[CR6] Apple M (2018). The struggle for democracy in education: Lessons from social realities.

[CR7] Azevedo, J. P. (2020). Learning poverty. World bank policy research working paper, Washington DC: World Bank

[CR8] Ball S (2016). Neoliberal education? Confronting the slouching beast. Policy Features in Education.

[CR9] Barrett B, Carter MR, Chavas JP (2019). The economics of poverty traps. C.

[CR10] Bourdieu P, Passeron JC (1990). Reproduction in education, society and culture. Sage.

[CR11] Bowles S, Gintis H (1976). Schooling in capitalist America.

[CR12] Bowles S, Gintis H (2002). Schooling in capitalist America revisited. Sociology of education.

[CR13] Brando N (2020). Children’s abilities, freedom, and the process of capability-formation. Journal of Human Development and Capabilities.

[CR14] Brando N, Schweiger G (2019). Philosophy and child poverty.

[CR15] Brehm, W. Unterhalter, E. and Oketch, M. 2021, States of emergency: Education in a time of Covid-19’ NORRAG special issue 06, September

[CR16] Burchardt T, Hick R (2018). Inequality, advantage and the capability approach. Journal of Human Development and Capabilities..

[CR17] Castro-Kemp S, Mahmud A (2021). School closures and returning to school: Views of parents of children with disabilities in england during the Covid-19 pandemic. Front. Educ..

[CR18] Chase E, Bantebya-Kyomuhendo G (2015). Poverty and shame.

[CR19] Chiappero-Martinetti, E., Osmani, S. and Qizilbash, M. (eds.) (2020) *The Cambridge Handbook of the Capability Approach.* Cambridge: Cambridge University Press.

[CR20] D’Agnese S (2017). Reclaiming education in the age of pisa. challenging OECD’s educational order.

[CR21] De Jaeghere J (2021). A capability pedagogy for excluded youth: Fostering recognition and imagining alternative futures. Education, Citizenship and Social Justice.

[CR22] Education in England (2020). Annual Report 2020—Education Policy Institute (epi.org.uk)

[CR23] Equal Education, 2002. https://equaleducation.org.za/2022/01/19/media-statement-a-raw-deal-matric-class-of-2021-seriously-hurt-by-the-rotational-timetabling-system/

[CR24] Ferrannini A, Barbieri E, Biggeri M, Di Tommaso MR (2021). Industrial policy for sustainable human development in the post-Covid19 era. World Development.

[CR25] Haberman M (2010). The pedagogy of poverty versus good teaching. Phi Delta Kappan.

[CR26] Hanes, D.W. (2019) Child poverty, impoverished parenting, and normative childhood: some words of caution. In: *Philosophy and child poverty: Reflections on the ethics and politics of poor children and their families* Eds. Brando N. and Schweiger G. 19–49, Cham: Springer International Publishing.

[CR27] Hart CS (2012). The capability approach and education. Cambridge Journal of Education.

[CR28] Hempel-Jorgensen, A. (2019) Inequality of pedagogy in english schools: How significant is it? BERA

[CR29] Hevia FJ, Vergara-Lope S, Velásquez-Durán A, Calderón D (2022). Estimation of the fundamental learning loss and learning poverty related to COVID-19 pandemic in Mexico. International Journal of Educational Development.

[CR30] Holt, L., and Murray, L. (2021). Children and Covid 19 in the UK. Children's Geographies, 1–8 10.1080/14733285.2021.1921699.

[CR31] Holt L, Murray L (2022). Children and Covid 19 in the UK. Children's Geographies.

[CR32] House of Commons Library (2021) Briefing Paper Number 7096, *Poverty in the UK: statistics*. UK Parliament

[CR33] Kim LE, Asbury K (2020). ‘Like a rug had been pulled from under you’: The impact of COVID-19 on teachers in England during the first six weeks of the UK lockdown. British Journal of Educational Psychology.

[CR34] Lister R (2004). Poverty.

[CR35] Martin, A., Partika, A., Johnson, A. D., Castle, S., Horm, D., & Tulsa SEED Study Team. (2022). Both sides of the screen: Predictors of parents’ and teachers’ depression and food insecurity during COVID-19-Related distance learning. Early childhood research quarterly. 10.1016/j.ecresq.2022.02.001 PMC8825345 35153375

[CR36] Mitchell, C., Moletsane, R., MacEntee, K., and de Lange, N. (2020). Participatory visual methodologies in self-study for social justice teaching: A reflexive eye. *International handbook of self-study of teaching and teacher education practices*, 683–712.

[CR37] Moletsane, R. and Mitchell, C. (2018). Researching Sexual Violence with Girls in Rural South Africa. *The Wiley Handbook on Violence in Education: Forms, Factors and Preventions*, 433. NJ: John Wiley & Sons, Inc.

[CR38] Moletsane, R. (2022). Using photovoice to enhance young women’s participation in addressing gender-based violence in higher education. *Comparative Education* 1–20.

[CR39] Moss G, Allen R, Bradbury A, Duncan S, Harmey S, Levy R (2020). Primary teachers’ experience of the COVID-19 lockdown—Eight key messages for policymakers going forward.

[CR40] Nussbaum M (2000). Women and human development: The capabilities approach.

[CR41] Nussbaum M (2011). Creating capabilities.

[CR42] Peters M, Besley T (2014). Children in crisis: Child poverty and abuse in New Zealand. Educational Philosophy and Theory.

[CR43] Pritchett, L. (2019). A review essay: The politics and governance of basic education: A tale of two South African provinces. *RISE Research on Improving Systems of Education, Insight Series. Oxford, UK: Blavatnik School of Government, University of Oxford. *https://www.riseprogramme*. org/publications/book-review-politics-governance-basic-education-south-african-provinces*.

[CR44] Raffo C, Dyson A, Gunter H, Hall D, Jones L, Kalambouka A (2009). Education and poverty: Mapping the terrain and making the links to educational policy. International Journal of Inclusive Education.

[CR45] Rawls J (1971). A theory of justice.

[CR46] Robeyns I (2017). Wellbeing, freedom and social justice: the capability approach re-examined.

[CR65] Robeyns I, Olsaretti S (2018). The capabilities approach. The Oxford handbook of distributive justice.

[CR47] Schweiger G, Graf G (2015). A philosophical examination of social justice and child poverty.

[CR48] Sen A (1985). Well-being, agency and freedom. The Dewey Lectures 1984. The Journal of Philosophy.

[CR49] Sen A (1992). Inequality reexamined.

[CR50] Sen A (1999). Development as freedom.

[CR51] Sen A (2009). The idea of justice.

[CR52] Sen, A. (2006) What do we want from a theory of justice?. *Journal of Philosophy*. CIII (5).

[CR53] Smith, W. and Benavot, A. (2021) Quality education and global learning metrics In *Education and international development. An introduction* Eds. McCowan T and Unterhalter E, 189–206. London: Bloomsbury.

[CR54] Taylor C (2004). Modern social imaginaries.

[CR55] Terzi L (2005). Beyond the dilemma of difference: The capability approach to disability and special educational needs. Journal of Philosophy of Education.

[CR56] Terzi, L. (2021) Child poverty and educational inequalities. Paper presented at the seminar series on Child Poverty and Education: Philosophical reflections. 25th March 2021.

[CR57] Thompson I, McNicholl J, Menter I (2016). Student teachers’ perceptions of poverty and educational achievement. Oxford Review of Education.

[CR58] Treanor M (2020). Child poverty. Aspiring to survive.

[CR59] UNDP, 2022, Human Development Report.

[CR60] UNESCO, 2021, When schools shut: Gendered effects of covid 19 school closures Paris: UNESCO https://unesdoc.unesco.org/ark:/48223/pf0000379270

[CR61] UNESCO, 2022, Evidence on the gendered effects of school closures. A systematic review Paris: UNESCO https://unesdoc.unesco.org/ark:/48223/pf0000380935

[CR62] Unterhalter E, Yates C, Makinda H, North A (2012). Blaming the poor: Constructions of marginality and poverty in the Kenyan education sector. Compare—A Journal of Comparative and International Education.

[CR63] Unterhalter E, Longlands H, Peppin Vaughan R (2022). Gender and intersecting inequalities in education: Reflections on a framework for measurement. Journal of Human Development and Capabilities.

[CR64] Venkatapuram S (2020). Human capabilities and pandemics. Journal of Human Development and Capabilities.

[CR66] Vizard P (2021). The conservative governments’ record on social policy from May 2015 to pre-COVID 2020: Policies, spending and outcomes. CASE Report, 136.

[CR67] Vizard, P and Hills, J. (ed.). The Conservative government's record on social policy from May 2015 to pre-COVID 2020: Policies, spending and outcomes. London: CASE (Centre for analysis of Social Exclusion), London School of Economics

[CR68] Walker M (2019). Defending the need for a foundational epistemic capability in education. Journal of Human Development and Capabilities.

[CR69] Walker M, Unterhalter E (2007). Amartya Sen's capability approach and social justice in education.

[CR70] Walker M, Boni A, Martinez-Vargas C, Cin M (2022). An Epistemological break: Redefining participatory research in capabilitarian scholarship. Journal of Human Development and Capabilities.

[CR71] Weidmann B, Allen R, Bibby D, Coe R, James L, Plaister N, Thomson D (2021). Covid-19 disruptions: Attainment gaps and primary school responses.

[CR72] Wolff J, Lever Annabelle, Poama Andrei (2018). Method in ethics and public policy: Applied philosophy versus engaged philosophy. The Routledge handbook of ethics and public policy.

[CR73] World Bank. 2021. studies of learning losses internationally https://www.worldbank.org/en/topic/education/publication/the-state-of-the-global-education-crisis-a-path-to-recovery?cq_ck=16385

[CR74] World Inequality Report 2022, (https://wir2022.wid.world/executive-summary/)

